# Uncontrolled eating and sensation-seeking partially explain the prediction of future binge drinking from adolescent brain structure

**DOI:** 10.1016/j.nicl.2023.103520

**Published:** 2023-09-30

**Authors:** Roshan Prakash Rane, Milena Philomena Maria Musial, Anne Beck, Michael Rapp, Florian Schlagenhauf, Tobias Banaschewski, Arun L.W. Bokde, Marie-Laure Paillère Martinot, Eric Artiges, Frauke Nees, Herve Lemaitre, Sarah Hohmann, Gunter Schumann, Henrik Walter, Andreas Heinz, Kerstin Ritter, IMAGEN consortium

**Affiliations:** a Charité – Universitätsmedizin Berlin, corporate member of Freie Universität Berlin and Humboldt-Universität zu Berlin, Einstein Center for Neurosciences Berlin, Charitéplatz 1, 10117 Berlin, Germany; b Charité – Universitätsmedizin Berlin, corporate member of Freie Universität Berlin and Humboldt-Universität zu Berlin, Department of Psychiatry and Neurosciences | CCM, Charitéplatz 1, 10117 Berlin, Germany; c Humboldt-Universität zu Berlin, Faculty of Life Sciences, Department of Psychology, Unter den Linden 6, 10099 Berlin, Germany; d Charité – Universitätsmedizin Berlin, corporate member of Freie Universität Berlin and Humboldt-Universität zu Berlin, Bernstein Center for Computational Neuroscience, Charitéplatz 1, 10117 Berlin, Germany; e Health and Medical University, Campus Potsdam, Faculty of Health, Olympischer Weg 1, 14471 Potsdam, Germany; f Social and Preventive Medicine, Department of Sports and Health Sciences, University of Potsdam, Potsdam, Germany; g Department of Child and Adolescent Psychiatry and Psychotherapy, Central Institute of Mental Health, Medical Faculty Mannheim, Heidelberg University, Square J5, 68159 Mannheim, Germany; h Discipline of Psychiatry, School of Medicine and Trinity College Institute of Neuroscience, Trinity College Dublin, Dublin, Ireland; i Institut National de la Santé et de la Recherche Médicale, INSERM U 1299 “Trajectoires développementales & psychiatrie”, University Paris-Saclay, CNRS; Ecole Normale Supérieure Paris-Saclay, Centre Borelli; Gif-sur-Yvette; and AP-HP. Sorbonne University, Department of Child and Adolescent Psychiatry, Pitié-Salpêtrière Hospital, Paris, France; j Institut National de la Santé et de la Recherche Médicale, INSERM U 1299 “Trajectoires développementales & psychiatrie”, University Paris-Saclay, CNRS; Ecole Normale Supérieure Paris-Saclay, Centre Borelli; Gif-sur-Yvette; and Psychiatry Department, EPS Barthélémy Durand, Etampes, France; k Institute of Cognitive and Clinical Neuroscience, Central Institute of Mental Health, Medical Faculty Mannheim, Heidelberg University, Square J5, Mannheim, Germany; l Institute of Medical Psychology and Medical Sociology, University Medical Center Schleswig Holstein, Kiel University, Kiel, Germany; m NeuroSpin, CEA, Université Paris-Saclay, F-91191 Gif-sur-Yvette, France; n Institut des Maladies Neurodégénératives, UMR 5293, CNRS, CEA, Université de Bordeaux, 33076 Bordeaux, France; o Department of Child and Adolescent Psychiatry, Psychotherapy and Psychosomatics, University Medical Center Hamburg-Eppendorf, Hamburg, Germany; p Charité – Universitätsmedizin Berlin, corporate member of Freie Universität Berlin and Humboldt-Universität zu Berlin, Department of Psychiatry and Neuroscience, Centre for Population Neuroscience and Stratified Medicine (PONS), Charitéplatz 1, 10117 Berlin, Germany; q Centre for Population Neuroscience and Precision Medicine (PONS), Institute for Science and Technology of Brain-inspired Intelligence (ISTBI), Fudan University, Shanghai, China

**Keywords:** Binge drinking, Machine learning, Confound detection, Addiction, Sensation-seeking, Eating behavior

## Abstract

Binge drinking behavior in early adulthood can be predicted from brain structure during early adolescence with an accuracy of above 70%. We investigated whether this accurate prospective prediction of alcohol misuse behavior can be explained by psychometric variables such as personality traits or mental health comorbidities in a data-driven approach.

We analyzed a subset of adolescents who did not have any prior binge drinking experience at age 14 (IMAGEN dataset, *n* = 555, 52.61% female). Participants underwent structural magnetic resonance imaging at age 14, binge drinking assessments at ages 14 and 22, and psychometric questionnaire assessments at ages 14 and 22. We derived structural brain features from T1-weighted magnetic resonance and diffusion tensor imaging. Using Machine Learning (ML), we predicted binge drinking (age 22) from brain structure (age 14) and used counterbalancing with oversampling to systematically control for 110 + variables from a wide range of social, personality, and other psychometric characteristics potentially associated with binge drinking. We evaluated if controlling for any variable resulted in a significant reduction in ML prediction accuracy.

Sensation-seeking (-13.98 ± 1.68%), assessed via the Substance Use Risk Profile Scale at age 14, and uncontrolled eating (-13.98 ± 3.28%), assessed via the Three-Factor-Eating-Questionnaire at age 22, led to significant reductions in mean balanced prediction accuracy upon controlling for them. Thus, sensation-seeking and binge eating could partially explain the prediction of future binge drinking from adolescent brain structure.

Our findings suggest that binge drinking and binge eating at age 22 share common neurobiological precursors discovered by the ML model. These neurobiological precursors seem to be associated with sensation-seeking at age 14. Our results facilitate early detection of increased risk for binge drinking and inform future clinical research in trans-diagnostic prevention approaches for adolescent alcohol misuse.

## 1 Introduction

Binge drinking is an alcohol misuse behavior which is defined as recurring alcohol consumption that leads to a blood alcohol concentration of 0.08% (NIAAA, 2023). This is typically achieved by consuming at least 4 or 5 drinks within two hours for women and men, respectively (NIAAA, 2023). Binge drinking is highly prevalent among adolescents and young adults (Crews et al., 2007). This consumption pattern during adolescence can increase the risk of developing alcohol use disorder and other substance use disorders later in life and has long-term detrimental effects across physical health, mental well-being, and social domains (Chung et al., 2018).

During adolescence, the human brain undergoes structural (Giedd, 2004, Lebel and Beaulieu, 2011) and functional (Gu et al., 2015) maturation. While maturation typically starts in sensory cortical areas, the prefrontal cortex and associated executive functions continue to develop until early adulthood (Constantinidis and Luna, 2019, Sharma et al., 2014). It has been suggested that this mismatch, in combination with other neurodevelopmental patterns, renders adolescents especially prone to participate in risk-taking behaviors such as binge drinking (Crone et al., 2016, Crone and Richard Ridderinkhof, 2011, Geier, 2013).

Conversely, during the particularly vulnerable period of adolescence (Crews et al., 2007), binge drinking has been linked with deficits in executive functioning, specifically in inhibitory control (Carbia et al., 2018). This aligns with findings of structural and functional alterations in prefrontal brain regions observed in studies such as (Pérez-García et al., 2022) along with documented enhancements in electroencephalographic activity related to inhibitory control processes among adolescent binge drinkers, as reported by (Almeida-Antunes et al., 2021). Collectively, these studies suggest that neural factors may underpin binge drinking behavior in adolescents. However, they do not definitively answer whether these neural alterations cause or result from binge drinking, leaving the temporal dynamics unresolved (Newcomb et al., 1981, Rao et al., 2017). To close this gap, we analyzed structural magnetic resonance imaging (sMRI) brain data of adolescents who have had no prior binge drinking experiences.

We can identify neural predictors of binge drinking using Machine Learning (ML) on longitudinal neuroimaging datasets which contain information about adolescent alcohol misuse. One such dataset is provided by the IMAGEN project (Mascarell Maričić et al., 2020). Three ML studies on the IMAGEN cohort found that differences in adolescent brains can predict binge drinking in the future (Quinlan et al., 2020, Schumann et al., 2010, Werle et al., 2021). Particularly, (Rane et al., 2022) demonstrated on a large sample (n = 1182) that binge drinking at age 22 can be predicted from sMRI features at age 14 with an accuracy of more than 70%.

Complex alcohol misuse behaviors such as binge drinking co-occur with other binge- and addiction-related behaviors (Castro-Calvo et al., 2021, Escrivá-Martínez et al., 2020, Ferriter and Ray, 2011, Pompili and Laghi, 2019, Wasserman et al., 2020), personality traits (Adan et al., 2017, Werle et al., 2021), and other psychometric characteristics (Williams et al., 2020). The aforementioned ML studies (Quinlan et al., 2020, Schumann et al., 2010, Werle et al., 2021) controlled for the participant’s sex and the site of data acquisition. However, they did not consider the effects of a wide range of psychometric characteristics on their model prediction. Psycho-social factors such as personality traits or mental health comorbidities might mediate the relationship between neurobiological predictors and binge drinking behavior. Investigating whether psychometric characteristics affect the model prediction accuracy may help us to understand binge drinking from a trans-diagnostic perspective (Morris et al., 2022).

Therefore, in the present study, we systematically assessed whether a wide range of psychometric characteristics influence the accuracy of an ML model which can prospectively predict binge drinking from brain structure in initially binge-drinking-naïve adolescents. Specifically, from the IMAGEN dataset, we extracted 131 variables belonging to six categories: alcohol-related, binge- and addiction-related, mental health comorbidity, general health, personality, social, biographic, and familial risk. Using a confound control method in a data-driven approach, we controlled for each of these psychometric variables in an ML model and assessed how the prediction accuracy changed (Rane et al., 2022). If a psychometric variable led to a significant reduction in accuracy upon controlling for it, this implied that the variable could partially explain the ML model prediction. The ML model we used is the support vector machine (SVM-rbf) model from (Rane et al., 2022) which predicts total lifetime binge drinking episodes (BDEs) by age 22 from sMRI features at age 14. We trained the model on a subsample of the IMAGEN cohort with no prior binge drinking experiences at age 14 (n = 477) as this allowed us to investigate the neurobiological predictors of binge drinking that precede effects from prior binge drinking experiences (Newcomb et al., 1981, Rao et al., 2017). The psychometric variables which reduced prediction accuracy when controlled for provide us with trans-diagnostic insights into the relationship between the brain structural alterations preceding an adolescent’s first binge drinking experience and their future binge drinking behavior.

## 2 Methods

The search for explanatory variables was formulated as a series of confound control experiments. Formally, the predictive ML model is represented as *f_model_: X*  → *y*, where *X* denotes structural brain features of adolescents at age 14, and *y* denotes the number of lifetime BDEs measured at age 22. If the ML model’s accuracy reduced significantly after controlling for a psychometric variable *c*, this implies *c* was driving the association *f_model_: X*  → *y* via a confounding pathway *X*  ← *c* → *y*. To control for *c*, we used counterbalancing with oversampling, where subjects are randomly sampled with replacement until values of *c* are equally distributed across all categories of *y* (Rao et al., 2017). This resampling precedure removes the relation between *c* → *y*, consequently removing the confounding pathway *X*  ← *c* → *y*. This confound control technique has been established as a superior method to other confound control methods, such as counterbalancing with undersampling and confound regression, in the prediction of binge drinking from sMRI features (Rane et al., 2022).

**Fig. 1:**
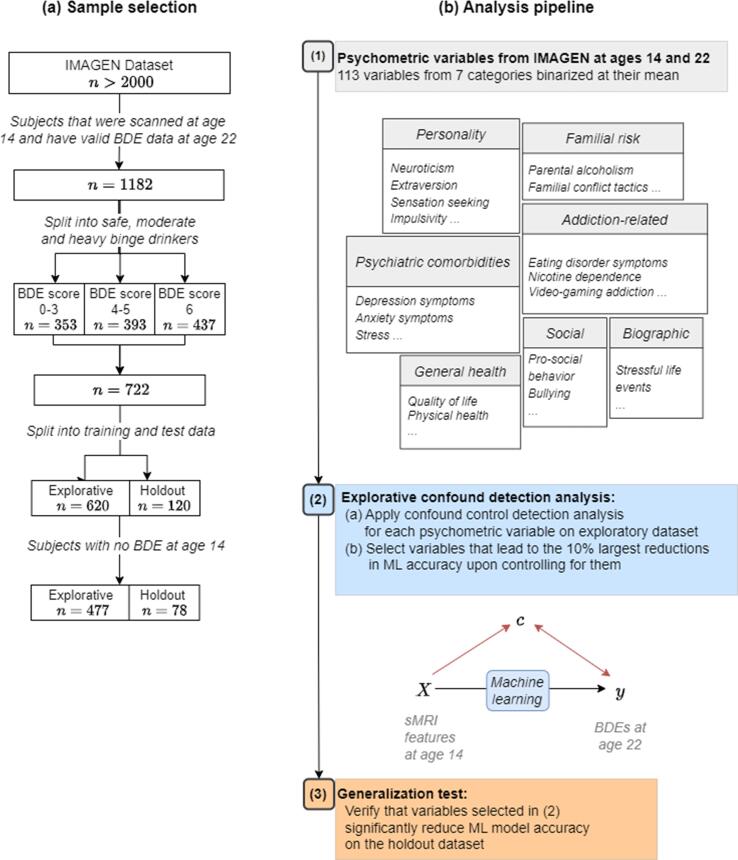
(a) Sample selection procedure. We included participants who had structural magnetic resonance imaging (sMRI) data at age 14, binge drinking episode (BDE) data at age 22, and a BDE score of < 4 (safe users) or of > 5 (heavy binge drinkers). From this, only the subset of participants with no prior BDEs at age 14 was selected. (b) The three stages of our analysis pipeline. ML, machine learning.

The analysis pipeline consisted of three stages (Fig. 1). In a first stage, we compiled a comprehensive list of 131 psychometric variables *c* potentially explaining the prediction of binge drinking *y* from MRI features *X*. The subsequent exploration stage served to identify the psychometric variables leading to the largest reductions in accuracy, while the generalization stage allowed us to test the significance of the effect of controlling for individual variables identified during the exploration stage in an independent sample.

### 2.1 Data

#### 2.1.1 Sample

All analyses are based on the IMAGEN dataset (Mascarell Maričić et al., 2020, Schuckit et al., 2017). The IMAGEN project is a longitudinal population-based study which followed-up more than 2000 adolescents and their parents across 4 time points over a period of up to 10 years. Participants were initially recruited in schools at 8 study sites across Europe and completed a variety of neuropsychological and neuroimaging measures, amongst others. More information about the IMAGEN sample, such as inclusion and exclusion criteria and neuroimaging protocols, are documented at https://imagen-project.org/. Out of the full dataset, 1182 adolescents were followed-up from age 14 to age 22. Since we were interested in brain features preceding binge drinking onset, we focused on subjects who had no prior BDEs at age 14. This sample was then divided into an exploratory dataset (*n* = 477) and a holdout dataset (*n* = 78). For sample characteristics and selection procedures, see Table 1 and Fig. 1. To explore generalizability, we repeated the analysis pipeline on a larger sample with maximum one BDE at age 14 (Supplementary Methods and Results).

**Table 1:** Sample characteristics.

	Exploratory dataset (*n* = 477)		Holdout dataset (*n* = 78)	
Baseline characteristic	Safe users (*n* = 254)	Heavy binge drinkers (*n* = 223)	Safe users (*n* = 39)	Heavy binge drinkers (*n* = 39)
Recruitment site				
Berlin, *n*	27	19	3	3
Nottingham, *n*	12	43	1	8
London, *n*	15	43	3	4
Paris, *n*	78	9	14	0
Dublin, *n*	7	33	1	9
Dresden, *n*	54	22	4	5
Hamburg, *n*	32	34	3	3
Mannheim, *n*	29	20	10	7
Sex				
Female, *n*	172	81	23	16
Male, *n*	82	142	16	23
AUDIT score at age 14, Mean ± *SD*	0.50 ± 0.87	0.77 ± 2.82	0.79 ± 1.67	0.41 ± 0.67
AUDIT score at age 22, Mean ± *SD*	2.98 ± 2.31	8.86 ± 4.01	2.98 ± 2.31	8.28 ± 4.62
ESPAD Alc last 12 months at age 14, Mean ± *SD*	1.35 ± 1.11	1.44 ± 1.26	1.35 ± 1.30	1.50 ± 1.21
ESPAD Alc last 12 months at age 22, Mean ± *SD*	2.98 ± 2.31	8.86 ± 4.01	3.71 ± 2.64	8.28 ± 4.62
TFEQ-R18 Uncontrolled eating FU3; *n* above/below	90 / 141	104 / 94	9 / 18	16 / 10
SURPS Sensation-seeking BL; *n* above/below mean	123 / 130	142 / 81	18 / 21	24 / 15

*Notes*. Alc, alcohol consumption occasions; AUDIT, Alcohol Use Disorder Identification Test (Saunders et al., 1993); ESPAD, European School Survey Project on Alcohol and other Drugs; SURPS, Substance Use Risk Profile Scale (Woicik et al., 2009); TFEQ-R18, Three-Factor Eating Questionnaire – Revised 18 (Karlsson et al., 2000).

#### 2.1.2 MRI features

Six hundred and fifty-six T1-weighted MRI features and 63 diffusion tensor imaging features were extracted per subject at age 14 and used as input for the ML algorithm. For details regarding sMRI data collection, preprocessing, and feature extraction, see Supplementary Methods.

#### 2.1.3 Binge drinking

The total lifetime number of BDEs by ages 14 and 22 was assessed via the European School Survey Project on Alcohol and other Drugs (ESPAD) questionnaire, wherein a BDE was defined as an occasion of being drunk from drinking alcoholic beverages. The number of BDEs was coded categorically on a scale from 0 to 6 (0 = no, 1 = one to two, 2 = three to five, 3 = six to nine, 4 = 10 to 19, 5 = 20 to 39, 6 = 40 or more BDEs). As described in Fig. 1a, subjects with information on binge drinking at age 22 were categorized into two groups of interest: safe users (ESPAD BDE score < 4, *n* = 353) and heavy binge-drinkers (ESPAD BDE score > 5, *n* = 437). Subjects with a score of 4 to 5 were dropped from all analyses.

#### 2.1.4 Psychometric variables

We extracted 131 psychometric variables from all questionnaires assessed via Psytools (Delosis Limited, Twickenham, UK) in adolescents at ages 14 and 22 as well as from family-history-relevant questionnaires answered by parents when adolescents were age 14 (Table S1, Supplementary Methods). Extracted variables were grouped into eight categories: alcohol-related, binge- and addiction-related, mental health comorbidity, general health, personality, social, biographic, and familial risk variables. Alcohol-related variables were included as a sanity check: If the confound detection method was effective, we would expect those variables to reduce the ML model’s accuracy upon controlling for them.

As counterbalancing with oversampling requires categorical variables *c* as input, all psychometric characteristics were binarized at their mean. If binarization resulted in any group containing less than 15% of cases, we excluded the respective variable from further analyses. Using this rule, in the exploration stage, counterbalancing was performed on 113 psychometric variables.

### 2.2 Analyses

We trained a non-linear SVM-rbf model to classify safe users from heavy binge-drinkers while controlling for sex and site (for details, see Rane et al., 2022). The mean balanced accuracy before (MBA_pre_) and after (MBA_post_) sequentially controlling for each psychometric variable were then compared to derive a measure of accuracy reduction (MBA_post-pre_; Supplementary Methods).

As shown in Fig. 1b, the confound detection analysis was performed in two stages: an exploration stage and a generalization stage. In the exploration stage, based on the distribution of MBA_post-pre_ in the exploratory dataset, we identified psychometric variables leading to the lowest ∼10% of MBA_post-pre_ values. In the generalization stage, we re-evaluated the variables identified during the exploration stage on an independent holdout dataset. The significance of MBA_post-pre_ per psychometric variable was determined using permutation tests (O’Halloran et al., 2017, Quinlan et al., 2020) (Supplementary Methods). To correct for multiple comparisons between empirical and permuted MBA_post-pre_ scores, Benjamini/Hochberg (non-negative) correction was applied.

## 3 Results

When not controlling for any psychometric variable, the ML model controlling for sex and site achieved MBA_pre_ = 67.35% (SD = 3.42%) on the exploratory dataset. On the holdout dataset, the model prospectively predicted binge drinking at age 22 from MRI features at age 14 with MBA_pre_ = 73.68% (SD = 1.14%; p =.001).

### 3.1 Exploration stage

**Fig. 2:**
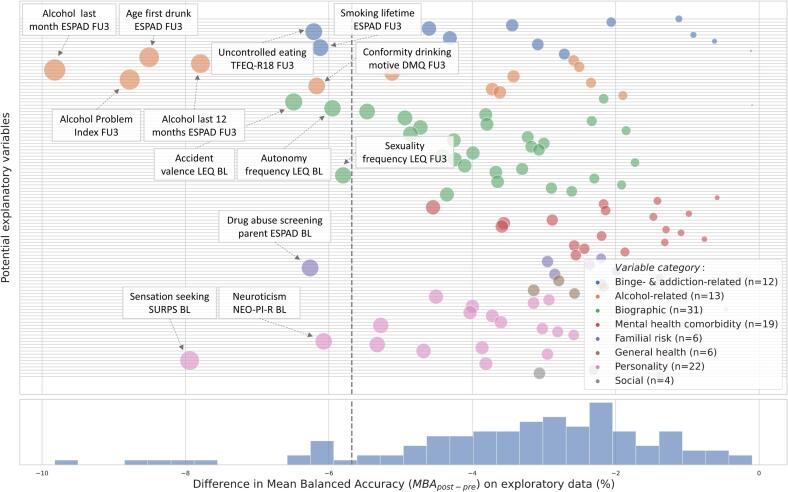
Exploration stage results: MBA_post-pre_ for psychometric variables in the exploratory dataset (*n* = 477). All values in percent. Vertical lines mark the threshold beyond which the lowest ∼10% of the MBA_post-pre_ values are located. For visualization purposes, dot size increases with increasing absolute MBA_post-pre_. For more information on psychometric variables, see Table S1. Alcohol Problem Index, Alcohol Problem Index (White and Labouvie, 1989); BL, assessed at adolescent’s age 14; ESPAD, European School Survey Project on Alcohol and other Drugs; FU3, assessed at adolescent's age 22; LEQ, Stressful Life Event Questionnaire (Newcomb et al., 1981); MBA_pre_ / MBA_post_, mean balanced accuracy before / after performing counterbalancing with oversampling for each psychometric variable; NEO-PI-R, Revised NEO Personality Inventory (Costa and McCrae, 1992); SURPS, Substance Use Risk Profile Scale (Woicik et al., 2009); TFEQ-R18, Three-Factor Eating Questionnaire – Revised 18 (Karlsson et al., 2000).

The reductions in accuracy obtained from additionally controlling for different psychometric variables are shown in Fig. 2 and Table S3. Alcohol-related variables descriptively led to the largest reductions in MBA_post_ compared to MBA_pre_. These variables served as a sanity check which confirmed that our method worked as expected. Besides alcohol-related variables, the variables leading to the lowest MBA_post-pre_ values belonged to the binge- and addiction-related, personality, biographic and familial risk categories. Thirteen variables from these categories crossed the ∼10% threshold and were taken to the generalization stage (for inter-correlations, see Table S4).

### 3.2 Generalization stage

**Fig. 3:**
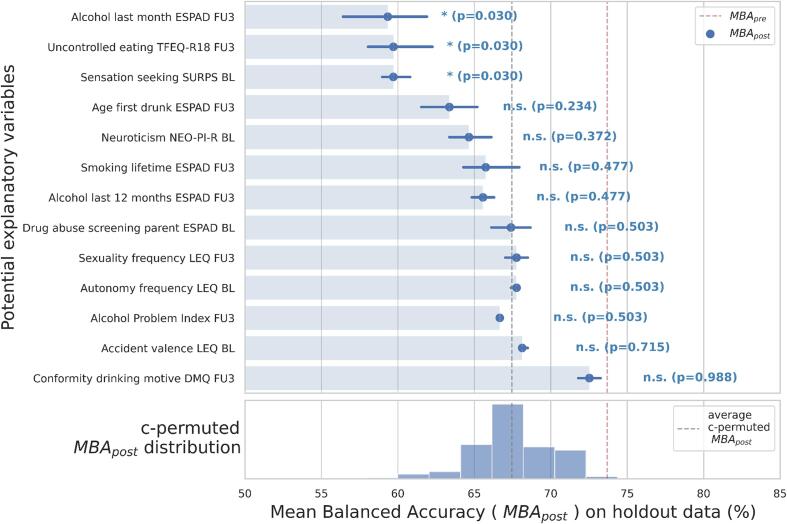
Generalization stage results: MBA_post_ for psychometric variables in the holdout dataset (*n* = 78). All values are in percent. Benjamini/Hochberg-corrected *p*-palues denote if the MBA_post-pre_ per psychometric variable was significantly different from the mean permuted MBA_post-pre_ (Table S1, Supplementary Methods). Alcohol Problem Index, Alcohol Problem Index (White and Labouvie, 1989); BL, assessed at adolescent’s age 14; ESPAD, European School Survey Project on Alcohol and other Drugs; FU3, assessed at adolescent's age 22; LEQ, Stressful Life Event Questionnaire (Newcomb et al., 1981); MBA_post_, mean balanced accuracy after performing counterbalancing with oversampling for each psychometric variable; NEO-PI-R, Revised NEO Personality Inventory (Costa and McCrae, 1992); SURPS, Substance Use Risk Profile Scale (Woicik et al., 2009); TFEQ, Three-Factor Eating Questionnaire – Revised 18 (Karlsson et al., 2000).

Out of the 13 psychometric variables selected from the exploration stage, only the reductions in accuracy caused by performing counterbalancing with oversampling on three variables reached significance (Fig. 3, Table S5). The first variable was alcohol consumption in the past month at age 22 according to the ESPAD questionnaire (MBA_post-pre_ = -14.34 ± 4.96%). Again, as alcohol-related variables were included as a sanity check, the significant reduction in accuracy upon controlling for this variable confirmed our method's validity. The remaining variables leading to significant reductions in accuracy upon controlling for were uncontrolled eating at age 22 (MBA_post-pre_ = -13.98 ± 3.28%) assessed via the Three Factor Eating Questionnaire – Revised 18 (TFEQ-R18) (Karlsson et al., 2000) and sensation-seeking at age 14 (MBA_post-pre_ = -13.98 ± 1.68%) assessed via the Substance Use Risk Profile Scale (SURPS) (Woicik et al., 2009).

## 4 Discussion

Our analysis revealed that two variables can partially explain the prospective prediction of binge drinking from brain structure: sensation-seeking (Woicik et al., 2009) at age 14 and uncontrolled eating (Karlsson et al., 2000) at age 22. This indicates that the neurobiological predictors of binge drinking are associated with sensation-seeking in early adolescence and with binge eating behavior in young adulthood. In 4.1, 4.2, we discuss the implications of each of these findings separately and relate them to the preexisting literature. In section 4.3, we make suggestions for future clinical research, before discussing limitations and conclusions in 4.4, 5.

### 4.1 Sensation-seeking

Sensation-seeking was measured using the ‘SURPS’ self-report questionnaire which assesses four personality characteristics potentially associated with substance abuse: impulsivity, anxiety sensitivity, hopelessness, and sensation-seeking (Woicik et al., 2009). Previous studies report that more pronounced sensation-seeking is associated with a higher likelihood of engaging in binge drinking (Adan et al., 2017, Werle et al., 2021). Interestingly, longitudinal studies found that sensation-seeking (McCabe et al., 2021) and within-person increases in sensation-seeking (Waddell and Chassin, 2023) during adolescence predict within-person increases in binge drinking during emerging adulthood.

Our results suggest that the structural neural features which are associated with sensation-seeking at age 14 predict heavy binge drinking behavior at age 22. This is principally in line with a cross-sectional study by (Holmes et al., 2016), who report associations between sensation-seeking, alcohol use frequency, and cortical thickness in areas associated with cognitive control. The association between sensation-seeking and binge drinking is strongest during late adolescence and subsequently weakens until young adulthood (Evans-Polce et al., 2018), hinting towards a potential role of brain maturation. Specifically, one might hypothesize that individuals with particularly slow or late onset of cortical maturation exhibit high sensation-seeking at age 14 and have a higher risk to engage in heavy binge drinking later in life (Constantinidis and Luna, 2019, Sharma et al., 2014).

### 4.2 Uncontrolled eating

Uncontrolled eating was measured using the self-report questionnaire ‘TFEQ-R18′ which assesses three facets of eating behavior: emotional eating, cognitive restraint, and uncontrolled eating (Anglé et al., 2009, Karlsson et al., 2000). Uncontrolled eating is conceptually related to binge eating, as it includes items such as ‘Do you go on eating binges though you are not hungry?’ (Karlsson et al., 2000). Several previous studies suggest associations between binge eating and binge drinking (Castro-Calvo et al., 2021, Escrivá-Martínez et al., 2020, Ferriter and Ray, 2011, Pompili and Laghi, 2019, Wasserman et al., 2020). Conceptually, both of these behaviors are characterized by repetitive over-consumption despite having conscious intentions to not engage in such behavior (Pompili and Laghi, 2019). Individuals engaging in both binge drinking and binge eating score high on impulsivity and sensation-seeking (Escrivá-Martínez et al., 2020, Ojala and Garriga, 2010) and report higher levels of negative urgency, i.e. the tendency to act out of negative emotional states (Van Swearingen and Noel, 2022).

Taken together with these previous studies, our results suggest that both binge drinking and binge eating share a common neurodevelopmental precursor. Specifically, the stage of cortical maturation at age 14 might be predictive of higher-order cognitive processing and executive functioning at age 22, in turn affecting both binge drinking and uncontrolled eating. Concomitantly, we found that future heavy binge-drinkers showed lower-than-average measures of brain structure, e. g. surface area or volume, in cortical association areas and higher-than-average measures in limbic regions at age 14 (Supplementary Methods and Results, Table S6). One could speculate that both binge behaviors are different manifestations of a common impulsive behavioral trait. Although self-reported impulsivity did not reduce the prediction accuracy of our ML analysis, self-report and behavioral measures capture different facets of impulsivity and are known to be independently related to substance use (Seo et al., 2019, Valente et al., 2023).

### 4.3 Clinical implications and future directions

Translated to clinical practice, our findings suggest that adolescents who are prone to heavy binge drinking might benefit from interventions affecting adolescent sensation-seeking. Approaches targeting sensation-seeking and other dimensions of the SURPS (Woicik et al., 2009) have indeed been successful in reducing binge drinking in adolescents, with effects lasting for up to three years (Edalati and Conrod, 2019). Importantly, such interventions might prevent not only binge drinking but also a wider range of impulsive binge behaviors such as binge eating (Flayelle and Lannoy, 2021). Trans-diagnostic approaches such as mindfulness-based interventions seem promising in reducing adolescent sensation-seeking as well as in preventing substance abuse (Najafi Chaleshtori et al., 2022, Narayanan and Naaz, 2018). However, the temporal and causal relations between brain structure, sensation-seeking, and binge behaviors remain to be investigated. Are individuals with brain structural risk factors receptive towards sensation-seeking-focused prevention approaches? Do the brain regions which are predictive of different binge behaviors overlap? These and other questions should be the focus of future longitudinal studies.

Another aspect that deserves special attention is sex- and gender-specific relations between brain structure, binge behaviors, and personality traits. In our sample, and according to previous research (Wilsnack et al., 2018), binge drinking was more common in male than in female adolescents. A similar pattern has been found for self-reported sensation-seeking (Cross et al., 2013). Binge eating is, however, more prevalent among women (Erskine and Whiteford, 2018) – a pattern we also descriptively observed in our dataset. This is in line with a higher prevalence of externalizing psychopathology, such as substance abuse, in male compared to female children and adolescents (Mayes et al., 2020, Mitchell et al., 2014, Spear, 2013). One might thus speculate that similar brain structural predispositions increase the probability of different binge behaviors depending on sex or gender. The role of sensation-seeking in predicting binge drinking and binge eating might be stronger in men than in women (Adan et al., 2017, Laghi et al., 2015). Hence, we recommend future studies to also consider sex- and gender-specific aspects in their analyses.

From a more methodological perspective, our confound-control method allowed us to explore the effect of a wide range of personal characteristics in ML settings. This strategy, potentially augmented by causal analysis techniques as in (Robert et al., 2020), can be an effective technique for explaining ML models used in predictive psychiatric applications. Identifying individual factors relevant to disease prediction can open up opportunities for patient subgroup identification and for individualized prevention recommendations in the sense of precision medicine (Quinlan et al., 2020).

### 4.4 Limitations

While our method helped us identify psychometric variables which explain the prediction of binge drinking from brain structure, it comes with several limitations. First, the counterbalancing with oversampling confound control method requires psychometric variables to be binarized. This can cause information loss. Nevertheless, (Rane et al., 2022) demonstrate that counterbalancing with oversampling is the most suitable technique when using non-linear ML algorithms as it controls for non-linear confounding components and does not reduce sample size.

Second, since we focus on binge-drinking-naïve adolescents, our sample size reduced to *n* = 555 from the original IMAGEN longitudinal cohort of *n* = 1182. This smaller sample size can affect the generalizability of our results. When repeating our analysis on a sample of adolescents with maximum one BDE at age 14 (*n* = 655), we could indeed not replicate our findings. However, not being able to replicate our findings in this sample does not necessarily indicate a lack of generalizability. On the contrary, factors explaining the binge drinking prediction in a non-binge-drinking-naïve subsample might be fundamentally different from those relevant in a binge-drinking-naïve subsample. This remains to be further investigated.

Lastly, while we considered an extensive set of psychometric variables available in the IMAGEN dataset, we recommend future studies to also consider additional relevant psychometrics such as eating behavior during early adolescence and early developmental trauma (Heinz et al., 2011, Saunders et al., 1993).

## 5 Conclusion

In conclusion, our findings highlight that the accurate prediction of binge drinking in young adulthood from adolescent brain structure can partially be explained by two variables: uncontrolled eating (Karlsson et al., 2000) at age 22 and sensation-seeking (Woicik et al., 2009) at age 14. This suggests that binge drinking shares common neural precursors with other binge behaviors exhibited in young adulthood and that these neural risk factors measured during adolescence coincide with sensation seeking. One possible explanation is that particularly slow or late onset of cortical maturation predisposes to impulsivity-related characteristics and behaviors, specifically to high sensation-seeking during adolescence (Wasserman et al., 2020) and to binge drinking and eating during young adulthood (Bø et al., 2016, Escrivá-Martínez et al., 2020). These relationships could inform clinical studies to test early interventions targeting sensation-seeking in adolescents with neural risk factors for future heavy binge drinking and binge eating.

## CRediT authorship contribution statement

**Roshan Prakash Rane:** Conceptualization, Methodology, Software, Data curation, Visualization, Writing – review & editing. **Milena Philomena Maria Musial:** Conceptualization, Formal analysis, Visualization, Writing – original draft. **Anne Beck:** Writing – review & editing. **Michael Rapp:** Writing – review & editing. **Florian Schlagenhauf:** Writing – review & editing, Funding acquisition. **Tobias Banaschewski:** Data curation, Writing – review & editing. **Arun L.W. Bokde:** Data curation, Writing – review & editing. **Marie-Laure Paillère Martinot:** Data curation, Writing – review & editing. **Eric Artiges:** Data curation, Writing – review & editing. **Frauke Nees:** Data curation, Writing – review & editing. **Herve Lemaitre:** Data curation, Writing – review & editing. **Sarah Hohmann:** Data curation, Writing – review & editing. **Gunter Schumann:** Data curation, Writing – review & editing. **Henrik Walter:** Writing – review & editing. **Andreas Heinz:** Writing – review & editing, Funding acquisition. **Kerstin Ritter:** Supervision, Project administration, Funding acquisition, Conceptualization, Writing – review & editing.

## Declaration of Competing Interest

Dr Banaschewski served in an advisory or consultancy role for eye level, Infectopharm, Lundbeck, Medice, Neurim Pharmaceuticals, Oberberg GmbH, Roche, and Takeda. He received conference support or speaker’s fee by Janssen, Medice and Takeda. He received royalities from Hogrefe, Kohlhammer, CIP Medien, Oxford University Press; the present work is unrelated to these relationships. Dr Barker has received honoraria from General Electric Healthcare for teaching on scanner programming courses. Dr Poustka served in an advisory or consultancy role for Roche and Viforpharm and received speaker’s fee by Shire. She received royalties from Hogrefe, Kohlhammer and Schattauer. The present work is unrelated to the above grants and relationships. The other authors declare that they have no known competing financial interests or personal relationships that could have appeared to influence the work reported in this paper.

## Data Availability

Data are available on request from the IMAGEN project at https://imagen-project.org/the-imagen-dataset/. Code can be found at https://github.com/RoshanRane/ML_for_IMAGEN.

## References

[b0005] Adan A., Forero D.A., Navarro J.F. (2017). Personality traits related to binge drinking: A systematic review. Frontiers in Psychiatry.

[b0010] Almeida-Antunes N., Crego A., Carbia C., Sousa S.S., Rodrigues R., Sampaio A., López-Caneda E. (2021). Electroencephalographic signatures of the binge drinking pattern during adolescence and young adulthood: A PRISMA-driven systematic review. NeuroImage Clin..

[b0015] Anglé S., Engblom J., Eriksson T., Kautiainen S., Saha M.-T., Lindfors P., Lehtinen M., Rimpelä A. (2009). Three factor eating questionnaire-R18 as a measure of cognitive restraint, uncontrolled eating and emotional eating in a sample of young finnish females. International Journal of Behavioral Nutrition and Physical Activity.

[b0020] Bø R., Billieux J., Landrø N.I. (2016). Which facets of impulsivity predict binge drinking?. Addictive Behaviors Reports.

[b0025] Carbia C., López-Caneda E., Corral M., Cadaveira F. (2018). A systematic review of neuropsychological studies involving young binge drinkers. Neuroscience and Biobehavioral Reviews.

[b0030] Castro-Calvo J., Cervigón-Carrasco V., Ballester-Arnal R., Giménez-García C. (2021). Cognitive processes related to problematic pornography use (PPU): A systematic review of experimental studies. Addictive Behaviors Reports.

[b0035] Chung T., Creswell K.G., Bachrach R., Clark D.B., Martin C.S. (2018). Adolescent binge drinking. Alcohol Res. Curr. Rev..

[b0045] Constantinidis C., Luna B. (2019). Neural substrates of inhibitory control maturation in adolescence. Trends in Neurosciences.

[b0050] Costa, P.T., McCrae, R.R., Psychological Assessment Resources, I., 1992. Revised NEO Personality Inventory (NEO PI-R) and NEO Five-Factor Inventory (NEO-FFI). Psychological Assessment Resources.

[b0055] Crews F., He J., Hodge C. (2007). Adolescent cortical development: A critical period of vulnerability for addiction. Adolesc. Drug Abuse Ment. Disord..

[b0060] Crone E.A., Richard Ridderinkhof K. (2011). The developing brain: from theory to neuroimaging and back. Developmental Cognitive Neuroscience.

[b0065] Crone E.A., van Duijvenvoorde A.C.K., Peper J.S. (2016). Annual research review: Neural contributions to risk-taking in adolescence – developmental changes and individual differences. Journal of Child Psychology and Psychiatry and Allied Disciplines (Cambridge).

[b0070] Cross C.P., Cyrenne D.-L.-M., Brown G.R. (2013). Sex differences in sensation-seeking: a meta-analysis. Scientific Reports.

[b0075] Edalati H., Conrod P.J. (2019). A review of Personality-Targeted interventions for prevention of substance misuse and related harm in community samples of adolescents. Frontiers in Psychiatry.

[b0080] Erskine H.E., Whiteford H.A. (2018). Epidemiology of binge eating disorder. Current Opinion in Psychiatry.

[b0085] Escrivá-Martínez T., Herrero R., Molinari G., Rodríguez-Arias M., Verdejo-García A., Baños R.M. (2020). binge eating and binge drinking: A Two-Way road? An integrative review. Current Pharmaceutical Design.

[b0090] Evans-Polce R.J., Schuler M.S., Schulenberg J.E., Patrick M.E. (2018). Gender- and age-varying associations of sensation seeking and substance use across young adulthood. Addictive Behaviors.

[b0095] Ferriter C., Ray L.A. (2011). binge eating and binge drinking: An integrative review. Eating Behaviors.

[b0100] Flayelle M., Lannoy S. (2021). Binge behaviors: Assessment, determinants, and consequences. Addictive Behaviors Reports.

[b0105] Geier C.F. (2013). Adolescent cognitive control and reward processing: Implications for risk taking and substance use. Horm. Behav. Puberty and Adolescence.

[b0110] Giedd J.N. (2004). Structural magnetic resonance imaging of the adolescent brain. Annals of the New York Academy of Sciences.

[b0115] Gu S., Satterthwaite T.D., Medaglia J.D., Yang M., Gur R.E., Gur R.C., Bassett D.S. (2015). Emergence of system roles in normative neurodevelopment. Proceedings of the National Academy of Sciences.

[b0120] Heinz A.J., Beck A., Meyer-Lindenberg A., Sterzer P., Heinz A. (2011). Cognitive and neurobiological mechanisms of alcohol-related aggression. Nature Reviews. Neuroscience.

[b0125] Holmes A.J., Hollinshead M.O., Roffman J.L., Smoller J.W., Buckner R.L. (2016). individual differences in cognitive control circuit anatomy link sensation seeking, impulsivity, and substance use. The Journal of Neuroscience.

[b0130] Karlsson J., Persson L.-O., Sjöström L., Sullivan M. (2000). Psychometric properties and factor structure of the Three-Factor eating questionnaire (TFEQ) in obese men and women. Results from the swedish Obese subjects (SOS) study. Int. J. Obes..

[b0135] Laghi F., Pompili S., Baumgartner E., Baiocco R. (2015). The role of sensation seeking and motivations for eating in female and male adolescents who binge eat. Eating Behaviors.

[b0140] Lebel C., Beaulieu C. (2011). Longitudinal development of human brain wiring continues from childhood into adulthood. The Journal of Neuroscience.

[b0145] Mascarell Maričić, L., Walter, H., Rosenthal, A., Ripke, S., Quinlan, E.B., Banaschewski, T., Barker, G.J., Bokde, A.L.W., Bromberg, U., Büchel, C., Desrivières, S., Flor, H., Frouin, V., Garavan, H., Itterman, B., Martinot, J.-L., Martinot, M.-L.P., Nees, F., Orfanos, D.P., Paus, Tomáš, Poustka, L., Hohmann, S., Smolka, M.N., Fröhner, J.H., Whelan, R., Kaminski, J., Schumann, G., Heinz, A., Albrecht, L., Andrew, C., Arroyo, M., Artiges, E., Aydin, S., Bach, C., Banaschewski, T., Barbot, A., Barker, G., Boddaert, N., Bokde, A., Bricaud, Z., Bromberg, U., Bruehl, R., Büchel, C., Cachia, A., Cattrell, A., Conrod, P., Constant, P., Dalley, J., Decideur, B., Desrivieres, S., Fadai, T., Flor, H., Frouin, V., Gallinat, J., Garavan, H., Briand, F.G., Gowland, P., Heinrichs, B., Heinz, A., Heym, N., Hübner, T., Ireland, J., Ittermann, B., Jia, T., Lathrop, M., Lanzerath, D., Lawrence, C., Lemaitre, H., Lüdemann, K., Macare, C., Mallik, C., Mangin, J.-F., Mann, K., Martinot, J.-L., Mennigen, E., de Carvahlo, F.M., Mignon, X., Miranda, R., Müller, K., Nees, F., Nymberg, C., Paillere, M.-L., Paus, Tomas, Pausova, Z., Poline, J.-B., Poustka, L., Rapp, M., Robert, G., Reuter, J., Rietschel, M., Ripke, S., Robbins, T., Rodehacke, S., Rogers, J., Romanowski, A., Ruggeri, B., Schmäl, C., Schmidt, D., Schneider, S., Schumann, M., Schubert, F., Schwartz, Y., Smolka, M., Sommer, W., Spanagel, R., Speiser, C., Spranger, T., Stedman, A., Steiner, S., Stephens, D., Strache, N., Ströhle, A., Struve, M., Subramaniam, N., Topper, L., Walter, H., Whelan, R., Williams, S., Yacubian, J., Zilbovicius, M., Wong, C.P., Lubbe, S., Martinez-Medina, L., Fernandes, A., Tahmasebi, A., IMAGEN consortium, 2020. The IMAGEN study: a decade of imaging genetics in adolescents. Mol. Psychiatry 25, 2648–2671. doi: 10.1038/s41380-020-0822-5. 10.1038/s41380-020-0822-5 PMC7577859 32601453

[b0150] Mayes S.D., Castagna P.J., Waschbusch D.A. (2020). Sex differences in externalizing and internalizing symptoms in ADHD, autism, and general population samples. Journal of Psychopathology and Behavioral Assessment.

[b0155] McCabe C.J., Wall T.L., Gonzalez M.R., Meruelo A.D., Eberson-Shumate S.C., Clark D.B., Nooner K.B., Brown S.A., Tapert S.F. (2021). Associations of developmental imbalance between sensation seeking and premeditation in adolescence and heavy episodic drinking in emerging adulthood. Alcoholism, Clinical and Experimental Research.

[b0160] Mitchell K.S., Wolf E.J., Reardon A.F., Miller M.W. (2014). Association of eating disorder symptoms with internalizing and externalizing dimensions of psychopathology among men and women. The International Journal of Eating Disorders.

[b0165] Morris S.E., Sanislow C.A., Pacheco J., Vaidyanathan U., Gordon J.A., Cuthbert B.N. (2022). Revisiting the seven pillars of RDoC. BMC Medicine.

[b0170] Najafi Chaleshtori M., Asgari P., Heidari A., Dasht Bozorgi Z., Hafezi F. (2022). Effectiveness of mindfulness-based stress reduction intervention in distress tolerance and sensation-seeking in adolescents with a drug-addicted parent. J. Res. Health.

[b0175] Narayanan, G., Naaz, S., 2018. A Transdiagnostic Approach to Interventions in Addictive Disorders- Third wave therapies and other current interventions. Indian J. Psychiatry 60, S522–S528. doi: 10.4103/psychiatry.IndianJPsychiatry_20_18. 10.4103/psychiatry.IndianJPsychiatry_20_18 PMC5844166 29540925

[b0180] National Institute on Alcohol Abuse and Alcoholism (NIAAA), 2023. Drinking Levels Defined | National Institute on Alcohol Abuse and Alcoholism (NIAAA) [WWW Document]. URL https://www.niaaa.nih.gov/alcohol-health/overview-alcohol-consumption/moderate-binge-drinking (accessed 9.10.23).

[b0185] Newcomb M.D., Huba G.J., Bentler P.M. (1981). A multidimensional assessment of stressful life events among adolescents: Derivation and correlates. Journal of Health and Social Behavior.

[b0190] O’Halloran L., Nymberg C., Jollans L., Garavan H., Whelan R. (2017). The potential of neuroimaging for identifying predictors of adolescent alcohol use initiation and misuse. Addiction.

[b0195] Ojala M., Garriga G.C. (2010). Permutation tests for studying classifier performance. Journal of Machine Learning Research.

[b0200] Oliva R., Morys F., Horstmann A., Castiello U., Begliomini C. (2019). The impulsive brain: Neural underpinnings of binge eating behavior in normal-weight adults. Appetite.

[b0205] Pérez-García J.M., Suárez-Suárez S., Doallo S., Cadaveira F. (2022). Effects of binge drinking during adolescence and emerging adulthood on the brain: A systematic review of neuroimaging studies. Neuroscience and Biobehavioral Reviews.

[b0210] Pompili S., Laghi F. (2019). binge eating and binge drinking among adolescents: the role of drinking and eating motives. Journal of Health Psychology.

[b0215] Prunell-Castañé A., Jurado M.Á., García-García I. (2021). Clinical binge eating, but not uncontrolled eating, is associated with differences in executive functions: Evidence from meta-analytic findings. Addictive Behaviors Reports.

[b0220] Quinlan, E.B., Banaschewski, T., Barker, G.J., Bokde, A.L.W., Bromberg, U., Büchel, C., Desrivières, S., Flor, H., Frouin, V., Garavan, H., Heinz, A., Brühl, R., Martinot, J.-L., Paillère Martinot, M.-L., Nees, F., Orfanos, D.P., Paus, T., Poustka, L., Hohmann, S., Smolka, M.N., Fröhner, J.H., Walter, H., Whelan, R., Schumann, G., IMAGEN Consortium, 2020. Identifying biological markers for improved precision medicine in psychiatry. Mol. Psychiatry 25, 243–253. doi: 10.1038/s41380-019-0555-5. 10.1038/s41380-019-0555-5 PMC6978138 31676814

[b0225] Rane, R.P., de Man, E.F., Kim, J., Görgen, K., Tschorn, M., Rapp, M.A., Banaschewski, T., Bokde, A.L., Desrivieres, S., Flor, H., Grigis, A., Garavan, H., Gowland, P.A., Brühl, R., Martinot, J.-L., Martinot, M.-L.P., Artiges, E., Nees, F., Papadopoulos Orfanos, D., Lemaitre, H., Paus, T., Poustka, L., Fröhner, J., Robinson, L., Smolka, M.N., Winterer, J., Whelan, R., Schumann, G., Walter, H., Heinz, A., Ritter, K., IMAGEN consortium, 2022. Structural differences in adolescent brains can predict alcohol misuse. eLife 11, e77545. doi: 10.7554/eLife.77545. 10.7554/eLife.77545 PMC9255959 35616520

[b0230] Rao A., Monteiro J.M., Mourao-Miranda J. (2017). Predictive modelling using neuroimaging data in the presence of confounds. NeuroImage.

[b0235] Robert, G.H., Luo, Q., Yu, T., Chu, C., Ing, A., Jia, T., Papadopoulos Orfanos, D., Burke-Quinlan, E., Desrivières, S., Ruggeri, B., Spechler, P., Chaarani, B., Tay, N., Banaschewski, T., Bokde, A.L.W., Bromberg, U., Flor, H., Frouin, V., Gowland, P., Heinz, A., Ittermann, B., Martinot, J.-L., Paillère Martinot, M.-L., Nees, F., Poustka, L., Smolka, M.N., Vetter, N.C., Walter, H., Whelan, R., Conrod, P., Barker, T., Garavan, H., Schumann, G., for the IMAGEN Consortium, 2020. Association of Gray Matter and Personality Development With Increased Drunkenness Frequency During Adolescence. JAMA Psychiatry 77, 409–419. doi: 10.1001/jamapsychiatry.2019.4063. 10.1001/jamapsychiatry.2019.4063 PMC6990803 31851304

[b0240] Saunders J.B., Aasland O.G., Babor T.F., De la Fuente J.R., Grant M. (1993). Development of the alcohol use disorders identification test (AUDIT): WHO collaborative project on early detection of persons with harmful alcohol consumption-II. Addiction.

[b0245] Schuckit M.A., Smith T.L., Danko G., Anthenelli R., Schoen L., Kawamura M., Kramer J., Dick D.M., Neale Z., Kuperman S., McCutcheon V., Anokhin A.P., Hesselbrock V., Hesselbrock M., Bucholz K. (2017). A prospective comparison of how the level of response to alcohol and impulsivity relate to future DSM-IV alcohol problems in the COGA youth panel. Alcoholism, Clinical and Experimental Research.

[b0250] Schumann G., Loth E., Banaschewski T., Barbot A., Barker G., Büchel C., Conrod P.J., Dalley J.W., Flor H., Gallinat J., Garavan H., Heinz A., Itterman B., Lathrop M., Mallik C., Mann K., Martinot J.-L., Paus T., Poline J.-B., Robbins T.W., Rietschel M., Reed L., Smolka M., Spanagel R., Speiser C., Stephens D.N., Ströhle A., Struve M. (2010). The IMAGEN study: reinforcement-related behaviour in normal brain function and psychopathology. Molecular Psychiatry.

[b0255] Seo S., Beck A., Matthis C., Genauck A., Banaschewski T., Bokde A.L.W., Bromberg U., Büchel C., Quinlan E.B., Flor H., Frouin V., Garavan H., Gowland P., Ittermann B., Martinot J.-L., Paillère Martinot M.-L., Nees F., Papadopoulos Orfanos D., Poustka L., Hohmann S., Fröhner J.H., Smolka M.N., Walter H., Whelan R., Desrivières S., Heinz A., Schumann G., Obermayer K. (2019). Risk profiles for heavy drinking in adolescence: differential effects of gender. Addiction Biology.

[b0260] Sharma L., Markon K.E., Clark L.A. (2014). Toward a theory of distinct types of “impulsive” behaviors: A meta-analysis of self-report and behavioral measures. Psychological Bulletin.

[b0265] Spear L.P. (2013). Adolescent neurodevelopment. J. Adolesc. Health, Emerging Issues in Adolescent Health.

[b0270] Valente J., Pietrobom T., Mihic J., Caetano S., Mari J., Sanchez Z.M. (2023). Externalizing and internalizing problems as predictors of alcohol-related harm and binge drinking in early adolescence: the role of gender. Journal of Affective Disorders.

[b0275] Van Swearingen K.M., Noel N.E. (2022). Impulsivity traits associated with disordered eating and binge drinking among female college students. Journal of American College Health.

[b0280] Waddell J.T., Chassin L. (2023). Multilevel longitudinal relations among impulsive traits, positive expectancies, and binge drinking from late adolescence to adulthood: A developmental test of acquired preparedness. Alcoholism, Clinical and Experimental Research.

[b0285] Wasserman A.M., Mathias C.W., Hill-Kapturczak N., Karns-Wright T.E., Dougherty D.M. (2020). The development of impulsivity and sensation seeking: Associations with substance use among At-Risk adolescents. Journal of Research on Adolescence.

[b0290] Werle D., Schroeder P.A., Wolz I., Svaldi J. (2021). incentive sensitization in binge behaviors: A mini review on electrophysiological evidence. Addictive Behaviors Reports.

[b0295] Whelan R., Watts R., Orr C.A., Althoff R.R., Artiges E., Banaschewski T., Barker G.J., Bokde A.L.W., Büchel C., Carvalho F.M., Conrod P.J., Flor H., Fauth-Bühler M., Frouin V., Gallinat J., Gan G., Gowland P., Heinz A., Ittermann B., Lawrence C., Mann K., Martinot J.-L., Nees F., Ortiz N., Paillère-Martinot M.-L., Paus T., Pausova Z., Rietschel M., Robbins T.W., Smolka M.N., Ströhle A., Schumann G., Garavan H. (2014). Neuropsychosocial profiles of current and future adolescent alcohol misusers. Nature.

[b0300] White H.R., Labouvie E.W. (1989). Towards the assessment of adolescent problem drinking. Journal of Studies on Alcohol.

[b0305] Williams G.C., Battista K., deGroh M., Jiang Y., Morrison H., Leatherdale S.T. (2020). Longitudinal associations between bullying and alcohol use and binge drinking among grade 9 and 10 students in the COMPASS study. Canadian Journal of Public Health.

[b0310] Wilsnack R.W., Wilsnack S.C., Gmel G., Kantor L.W. (2018). Gender differences in binge drinking. Alcohol Res. Curr. Rev..

[b0315] Woicik P.A., Stewart S.H., Pihl R.O., Conrod P.J. (2009). The substance use risk profile scale: A scale measuring traits linked to reinforcement-specific substance use profiles. Addictive Behaviors.

