# Supplementary material for: Uncontrolled eating and sensation-seeking partially explain the prediction of future binge drinking from adolescent brain structure
Source: Neuroimage Clin. 2023 Sep 30;40:103520. doi: 10.1016/j.nicl.2023.103520 (PMC10585345; doi:10.1016/j.nicl.2023.103520)
Supplement: Supplementary Material [file mmc1.docx]

# Supplementary Material

for

Uncontrolled eating and sensation-seeking partially explain the prediction of binge drinking from adolescent brain structure

# SUPPLEMENTARY METHODS

## Data

### MRI features

The MRI features used as input for the SVM model have previously been described in Rane et al. (Rane et al., 2022). T1-weighted structural data were collected using the ADNI protocol (Wyman et al., 2013) in 3-tesla scanners. Preprocessing was performed using the Free Surfer recon-all pipeline and included normalization to the Talairach template and segmentation into grey matter, white matter, and cerebrospinal fluid. The features of grey matter volume (in mm3), thickness (in mm), surface area (in mm2), and surface curvature were extracted for 34 cortical regions per hemisphere based on the Desikan-Killiany atlas. For 45 sub-cortical regions, mean intensity and volume were extracted. Additional global features included total grey matter, total white matter, total CSF, and total intracranial volumes.

DTI images were collected using the protocol by Jones et al. (Jones et al., 2002). Fractional anisotropy (DTI-FA) images derived using FMRIB’s Diffusion Toolbox FDT (FMRIB, Oxford, UK) were normalized to the MNI152 template. For 63 white matter-including brain regions, the average DTI-FA intensity was calculated using the TBSS toolbox (Smith et al., 2006). DTI- FA intensity greater than 3 standard deviations from the mean was considered an outlier criterion.

### Potential explanatory variables

If summary variables used for our confound control strategy were not pre-calculated and provided in the raw IMAGEN datafiles of respective Psytools (Delosis Limited, Twickenham, UK) questionnaires, we computed them based on the IMAGEN documentation (<https://imagen-project.org/>). According to this documentation, missing values in Psytools instrument variables of subjects included in the dataset per questionnaire represent skipped questions due to applied jump rules. For subjects included in the raw dataset per questionnaire, we thus imputed missing values in items included in the summary variable with the value indicating a low or arbitrary level of the measured construct. Missing values in summary variables were equally imputed with the value indicating a low or arbitrary level of the respective construct. Imputation values for all items and summary variables can be found in Table S1. If subjects were not included in the raw dataset of a Psytools instrument, this indicated that these subjects did not fill out the respective questionnaire. For these subjects, missing summary variable values were imputed using nearest neighbor imputation.

Binarized potential explanatory variables with less than 15% of cases in any group were excluded from further analyses as ML models trained with counterbalancing with oversampling might not fit the data reliably when there is a disproportionately large number of duplicate samples in one classification group (Menardi and Torelli, 2014).

### Sample with maximum one prior BDE at age 14

To explore the generalizability of our findings, we repeated the exploratory and generalization stage analyses on a sample with maximum one BDE at age 14. This sample included subjects with no prior BDEs at age 14 and is thus not independent of our sample of primary interest.

**Table S2.** Sample characteristics for subjects with maximum one prior BDE at age 14.

|  | **Exploratory dataset (*n* = 565)** | | **Holdout dataset (*n* = 90)** | |
| --- | --- | --- | --- | --- |
| **Baseline characteristic** | **Safe users**  **(*n* = 275)** | **Heavy binge drinkers**  **(*n* = 290)** | **Safe users (*n* = 41)** | **Heavy binge drinkers (*n* = 49)** |
| Recruitment site |  |  |  |  |
| Berlin, *n* | 29 | 26 | 3 | 5 |
| Nottingham, *n* | 15 | 63 | 1 | 1 |
| London, *n* | 16 | 58 | 3 | 3 |
| Paris, *n* | 88 | 11 | 15 | 15 |
| Dublin, *n* | 7 | 39 | 1 | 1 |
| Dresden, *n* | 56 | 27 | 4 | 6 |
| Hamburg, *n* | 33 | 43 | 4 | 4 |
| Mannheim, *n* | 31 | 23 | 10 | 10 |
| Sex |  |  |  |  |
| Female, *n* | 187 | 111 | 24 | 22 |
| Male, *n* | 88 | 179 | 17 | 27 |
| AUDIT score at age 14, Mean ± *SD* | 0.68 ± 1.21 | 1.26 ± 2.89 | 0.76 ± 1.64 | 1.10 ± 1.78 |
| AUDIT score at age 22, Mean ± *SD* | 2.98 ± 2.26 | 8.92 ± 4.15 | 3.56 ± 2.67 | 8.61 ± 4.81 |
| ESPAD Alc last 12 months at age 14, Mean ± *SD* | 1.42 ± 1.17 | 1.73 ± 1.35 | 1.30 ± 1.29 | 1.62 ± 1.16 |
| ESPAD Alc last 12 months at age 22, Mean ± *SD* | 2.98 ± 2.26 | 8.92 ± 4.15 | 3.56 ± 2.67 | 8.61 ± 4.81 |
| TFEQ-R18 Uncontrolled eating FU3; *n* above/below (missing for 69 subjects) | 98/149 | 141/119 | 16/18 | 28/17 |
| SURPS Sensation-seeking BL; *n* above/below mean (missing for 1 subject) | 134/140 | 190/100 | 19/22 | 30/19 |

*Notes*. Alc, alcohol consumption occasions; AUDIT, Alcohol Use Disorder Identification Test (Saunders et al., 1993); ESPAD, European School Survey Project on Alcohol and other Drugs; SURPS, Substance Use Risk Profile Scale (Woicik et al., 2009); TFEQ-R18, Three-Factor Eating Questionnaire – Revised 18 (Karlsson et al., 2000).

## Analyses

ML analyses were conducted as described in our previous study by Rane and colleagues (Rane et al., 2022).We used a non-linear support vector machine with radial basis function kernel (SVM-rbf) model as it was shown to outperform other linear and non-linear ML models (Rane et al., 2022). The SVM-rbf was trained to classify safe users from heavy binge-drinkers using a 7-fold outer cross-validation (CV) while controlling for sex and site (Rane et al., 2022) using counterbalancing with oversampling. A balanced accuracy (BA), robust against class imbalances in *y* (Rane et al., 2022), was obtained for each of the seven CV runs as the mean of the model’s accuracy at predicting class ‘safe users’ and class ‘heavy binge-drinkers’. The mean BA was obtained by averaging the BA across the seven CV runs and is denoted as MBA_pre_. We then retrained the ML model while controlling for sex, site, and sequentially for each psychometric variable using counterbalancing with oversampling. The mean BAs obtained after controlling for each additional variable are denoted as MBA_post_. A decrease in accuracy from MBA_pre_ to MBA_post_ is denoted by a negative MBA_post-pre_.

### Generalization stage

The significance of MBA_pre_ was determined using a permutation test procedure (Ojala and Garriga, 2010; Rane et al., 2022), wherein the ML algorithm was trained on the exploratory dataset with randomly shuffled labels *y* and tested on the unshuffled holdout data. This procedure was repeated 1000 times. The resulting distribution of permuted MBA_pre_ was then used as a standard of comparison for the empirical MBA_pre_: To derive *p*-values, we divided the number of absolute, mean-centered, permuted MBA_pre_ scores larger than the absolute, mean-centered, empirical MBA_pre_ score by the number of permutations, i. e. 1000.

To determine the significance of the MBA_post-pre_ for each of the selected potential explanatory variables during the generalization stage, we performed a permutation test. Specifically, 1000 randomly generated binary variables were entered into our confound control pipeline to generate 1000 permuted MBA_post_ scores on the BL-none-BDE holdout data. The MBA_post_ of each potential explanatory variable taken to the generalization stage was compared to the distribution of the MBA_post_ resulting from performing counterbalancing with oversampling on randomly generated variables. To derive *p*-values, we divided the number of absolute, mean-centered, permuted MBA_post_ scores lower than the absolute, mean-centered MBA_post_ score of the respective potential explanatory variable by the number of permutations, i. e. 1000. Please note that determining the significance of MBA_post_ as described above is mathematically equivalent to determining the significance of MBA_post-pre_ per psychometric variable.

### Informative brain features

We determined the most informative structural brain features when predicting BDEs in the holdout dataset using the SHAP (Shapley Additive exPlanations) technique (Lundberg and Lee, 2017; Rane et al., 2022). A higher average absolute SHAP value for a feature indicates a higher importance of that specific feature for the prediction of interest (Lundberg and Lee, 2017). Features with SHAP values two times higher than the average absolute SHAP value across all features are selected as most informative brain features. To deal with problematic correlations among features (Molnar, 2022), as in Rane et al. (Rane et al., 2022), we define only such sMRI features as significant which have SHAP values two times higher than the average absolute SHAP value across all features in at least 6 out of the 7 CV runs in the generalization stage. We also determine whether the informative features were higher or lower in heavy binge-drinkers compared to safe users.

# SUPPLEMENTARY RESULTS

## Exploration stage

**Table S3.** Exploration stage results: psychometric variables leading to the lowest ~10% of MBA_post-pre_ in exploratory dataset (*n* = 477).

| **Psychometric variable** | **Category** | **MBA_post_** | **SD_post_** | **MBA_(post-pre)_** | **SD(_post-pre)_** |
| --- | --- | --- | --- | --- | --- |
| Alcohol last month ESPAD FU3 | Alcohol-related | 57.52 | 2.39 | -9.82 | 3.08 |
| Alcohol Problem Index FU3 | Alcohol-related | 58.57 | 4.24 | -8.78 | 4.30 |
| Age first drunk ESPAD FU3 | Alcohol-related | 58.84 | 7.59 | -8.51 | 5.11 |
| Sensation-seeking SURPS BL | Personality | 59.40 | 4.58 | -7.94 | 4.22 |
| Alcohol last 12 months ESPAD FU3 | Alcohol-related | 59.55 | 7.10 | -7.79 | 5.29 |
| Accident valence LEQ BL | Biographic | 60.86 | 4.74 | -6.49 | 1.78 |
| Drug abuse screening parent ESPAD BL | Familial risk | 61.08 | 6.07 | -6.26 | 3.57 |
| Uncontrolled eating TFEQ-R18 FU3 | Binge- and addiction-related | 61.14 | 7.60 | -6.21 | 5.74 |
| Conformity drinking motive DMQ FU3 | Alcohol-related | 61.18 | 5.54 | -6.17 | 3.67 |
| Smoking lifetime ESPAD FU3 | Binge- and addiction-related | 61.22 | 8.14 | -6.12 | 5.90 |
| Neuroticism NEO-PI-R | Personality | 61.27 | 5.30 | -6.07 | 3.82 |
| Autonomy frequency LEQ BL | Biographic | 61.40 | 5.88 | -5.95 | 3.20 |
| Sexuality frequency LEQ FU3 | Biographic | 61.54 | 4.48 | -5.80 | 2.02 |

*Notes*. All values in percent. For more information on psychometric variables, see Table S1. Alcohol Problem Index, Alcohol Problem Index (White and Labouvie, 1989); BL, assessed at adolescent’s age 14; DMQ, Drinking Motives Questionnaire Revised (Cooper, 1994); ESPAD, European School Survey Project on Alcohol and other Drugs; FU3, assessed at adolescent's age 22; LEQ, Stressful Life Event Questionnaire (Newcomb et al., 1981); MBA_pre/post_, mean balanced accuracy before / after performing *counterbalancing with oversampling* for each variable; NEO-PI-R, Revised NEO Personality Inventory (Costa et al., 1992); SURPS, Substance Use Risk Profile Scale (Woicik et al., 2009); TFEQ-R18, Three-Factor Eating Questionnaire – Revised 18 (Karlsson et al., 2000).

**Table S4.** Chi-square tests for independence between binary psychometric variables in the exploratory dataset (*n* = 477).

|  | **Alcohol last month ESPAD FU3** | **Alcohol Problem Index FU3** | **Age first drunk ESPAD FU3** | **Sensation-seeking SURPS BL** | **Alcohol last 12 months ESPAD FU3** | **Accident valence LEQ BL** | **Drug abuse screening parent ESPAD BL** | **Uncontrolled eating TFEQ-R18 FU3** | **Conformity drinking motive DMQ FU3** | **Smoking lifetime ESPAD FU3** | **Neuroticism NEO-PI-R** | **Autonomy frequency LEQ BL** | **Sexuality frequency LEQ FU3** | **Binge drinking ESPAD FU3 (*y*)** |
| --- | --- | --- | --- | --- | --- | --- | --- | --- | --- | --- | --- | --- | --- | --- |
| **Alcohol last month ESPAD FU3** | 1.00 | 58.19*** | 58.61*** | 0.08 | 43.23*** | 1.26 | 17.39*** | 0.69 | 47.77*** | 0.08 | 0.08 | 0.03 | 52.69*** | 3.37 |
| **Alcohol Problem Index FU3** | 58.19*** | 1.00 | 212.14*** | 43.40*** | 180.31*** | 33.57*** | 9.04** | 58.49*** | 0.37 | 50.66*** | 43.40*** | 44.57*** | 200.10*** | 74.41*** |
| **Age first drunk ESPAD FU3** | 58.61*** | 212.14*** | 1.00 | 62.21*** | 1.04 | 77.16*** | 146.95*** | 45.25*** | 232.46*** | 53.33*** | 62.21*** | 60.67*** | 0.14 | 32.37*** |
| **Sensation-seeking SURPS BL** | 0.08 | 43.40*** | 62.21*** | 1.00 | 46.96*** | 0.71 | 15.05*** | 1.22 | 43.65*** | 0.31 | 0.00 | 0.01 | 56.82*** | 4.45* |
| **Alcohol last 12 months ESPAD FU3** | 43.23*** | 180.31*** | 1.04 | 46.96*** | 1.00 | 60.16*** | 122.22*** | 32.64*** | 199.61*** | 39.51*** | 47.15*** | 45.82*** | 0.42 | 21.9*** |
| **Accident valence LEQ BL** | 1.26 | 33.57*** | 77.16*** | 0.71 | 60.16*** | 1.00 | 9.04** | 3.74 | 32.40*** | 1.92 | 0.70 | 0.86 | 70.15*** | 8.62** |
| **Drug abuse screening parent ESPAD BL** | 17.39*** | 9.04** | 146.95*** | 15.05*** | 122.22*** | 9.04** | 1.00 | 22.96*** | 6.46* | 18.08*** | 13.78*** | 14.45*** | 126.80*** | 33.42*** |
| **Uncontrolled eating TFEQ-R18 FU3** | 0.69 | 58.49*** | 45.25*** | 1.22 | 32.64*** | 3.74 | 22.96*** | 1.00 | 61.25*** | 0.31 | 1.24 | 1.04 | 41.23*** | 1.02 |
| **Conformity drinking motive DMQ FU3** | 47.77*** | 0.37 | 232.46*** | 43.65*** | 199.61*** | 32.40*** | 6.46* | 61.25*** | 1.00 | 43.07*** | 36.38*** | 37.45*** | 184.61*** | 65.21*** |
| **Smoking lifetime ESPAD FU3** | 0.08 | 50.66*** | 53.33*** | 0.31 | 39.51*** | 1.92 | 18.08*** | 0.31 | 43.07*** | 1.00 | 0.31 | 0.21 | 48.72*** | 2.43 |
| **Neuroticism NEO-PI-R** | 0.08 | 43.40*** | 62.21*** | 0.00 | 47.15*** | 0.70 | 13.78*** | 1.24 | 36.38*** | 0.31 | 1.00 | 0.01 | 56.82*** | 4.45* |
| **Autonomy frequency LEQ BL** | 0.03 | 44.57*** | 60.67*** | 0.01 | 45.82*** | 0.86 | 14.45*** | 1.04 | 37.45*** | 0.21 | 0.01 | 1.00 | 55.43*** | 4.08* |
| **Sexuality frequency LEQ FU3** | 52.69*** | 200.10*** | 0.14 | 56.82*** | 0.42 | 70.15*** | 126.80*** | 41.23*** | 184.61*** | 48.72*** | 56.82*** | 55.43*** | 1.00 | 28.33*** |
| **Binge drinking ESPAD FU3 (*y*)** | 3.37 | 74.41*** | 32.37*** | 4.45* | 21.9*** | 8.62** | 33.42*** | 1.02 | 65.21*** | 2.43 | 4.45* | 4.08* | 28.33*** | 1 |

*Notes*. Values are *χ*^2^ with *df* = 1. Alcohol Problem Index, Alcohol Problem Index (White and Labouvie, 1989); BL, assessed at adolescent’s age 14; DMQ, Drinking Motives Questionnaire Revised (Cooper, 1994); ESPAD, European School Survey Project on Alcohol and other Drugs; FU3, assessed at adolescent's age 22; LEQ, Stressful Life Event Questionnaire (Newcomb et al., 1981); NEO-PI-R, Revised NEO Personality Inventory (Costa et al., 1992); SURPS, Substance Use Risk Profile Scale (Woicik et al., 2009); TFEQ, Three-Factor Eating Questionnaire – Revised 18 (Karlsson et al., 2000).

***, p<.001; **, p<.01; *, p<.05

## Generalization stage

| **Psychometric variable** | **Category** | **MBA_post_** | **SD_post_** | **MBA_(post-pre)_** | **SD_(post-pre)_** |
| --- | --- | --- | --- | --- | --- |
| Alcohol last month ESPAD FU3 | Alcohol-related | 59.34 | 4.42 | -14.34 | 4.96 |
| Uncontrolled eating TFEQ-R18 FU3 | Binge- and addiction-related | 59.71 | 3.30 | -13.98 | 3.28 |
| Sensation-seeking SURPS BL | Personality | 59.71 | 1.25 | -13.98 | 1.68 |
| Age first drunk ESPAD FU3 | Alcohol-related | 63.37 | 3.13 | -10.31 | 3.22 |
| Neuroticism NEO-PI-R BL | Personality | 64.65 | 1.94 | -9.03 | 2.56 |
| Alcohol last 12 months ESPAD FU3 | Alcohol-related | 65.57 | 1.15 | -8.12 | 1.94 |
| Smoking lifetime ESPAD FU3 | Binge- and addiction-related | 65.75 | 2.74 | -7.93 | 3.38 |
| Alcohol Problem Index FU3 | Alcohol-related | 66.67 | 0.00 | -7.02 |  |
| Drug abuse screening parent ESPAD BL | Familial risk | 67.40 | 1.94 | -6.28 | 2.63 |
| Sexuality frequency LEQ FU3 | Biographic | 67.77 | 1.15 | -5.92 | 1.84 |
| Autonomy frequency LEQ BL | Biographic | 67.77 | 0.48 | -5.92 | 1.22 |
| Accident valence LEQ BL | Biographic | 68.13 | 0.48 | -5.55 | 1.06 |
| Conformity drinking motive DMQ FU3 | Alcohol-related | 72.53 | 1.25 | -1.16 | 1.68 |

**Table S5.** Generalization stage results: MBA_post-pre_ for selected psychometric variables in the holdout dataset (*n* = 78).

*Notes*. All values in percent. For more information on psychometric variables, see Table S1. Alcohol Problem Index, Alcohol Problem Index (White and Labouvie, 1989); BL, assessed at adolescent’s age 14; DMQ, Drinking Motives Questionnaire Revised (Cooper, 1994); ESPAD, European School Survey Project on Alcohol and other Drugs; FU3, assessed at adolescent's age 22; LEQ, Stressful Life Event Questionnaire (Newcomb et al., 1981); MBA_pre/post_, mean balanced accuracy before / after performing *counterbalancing with oversampling* for each variable; NEO-PI-R, Revised NEO Personality Inventory (Costa et al., 1992); SURPS, Substance Use Risk Profile Scale (Woicik et al., 2009); TFEQ, Three-Factor Eating Questionnaire – Revised 18 (Karlsson et al., 2000).

## Informative brain features

Nineteen sMRI features were identified as most informative features for the prediction of binge drinking at age 22 from sMRI features at age 14 in BDE-naïve subjects at age 14 (Table S2). Out of these, 17 were derived from T1-weighted imaging and 2 from diffusion tensor imaging. Heavy binge drinkers exhibited lower fractional anisotropy in corpus callosum, which has been associated with cognitive impairments in young adult binge drinkers (Smith et al., 2017) as well as with relapse in abstinent subjects with AUD (Zou et al., 2018), as well as in posterior corona radiata compared to safe users. The T1-weighted structural brain features identified as most informative were largely located in cortical regions (15 features) as opposed to subcortical structures (2 features). Future heavy binge drinkers showed lower-than-average values across cortical association areas such as the secondary visual cortex (cuneus), the secondary somatosensory cortex (bank of the superior lateral sulcus), the temporal pole, a region associated with higher-order cognitive functions such as memory, semantic and emotional processing (Herlin et al., 2021), and Broca’s area (pars opercularis), associated with speech production (Lorca-Puls et al., 2021). Informative cortical brain features with higher-than-average values in future heavy binge drinkers compared to safe users were located in cortical areas associated with the integration of visual, spatial, and temporal perception and attention (superior- and inferiorparietal cortex) (Humphreys et al., 2020; Johns, 2014), and in limbic regions such as the isthmus cingulate, parahippocampal, and entorhinal cortices. The most common structural alterations were a reduction in volume or an increase in surface area in future heavy binge drinkers. Most informative subcortical alterations were found in the inferiorlateral ventricle and the pallidum, part of the basal ganglia and involved in motor control and reward processing (Smith et al., 2009).

**Table S6.** Most informative structural brain features for the prediction of binge drinking in the exploratory sample with no BDEs at age 14 (*n* = 477).

| **Imaging modality** | **Lower-than-average values in heavy binge-drinkers** | **Higher-than-average values in heavy binge-drinkers** |
| --- | --- | --- |
| T1w subcortical | Left inferiorlateral ventricle (volume) | Right pallidum (volume) |
| T1w cortical | Right cuneus (thickness and volume) | Left superiorparietal cortex (thicknessstd) |
|  | Left temporal pole (thicknessstd) | Right superiorparietal cortex (thicknessstd) |
|  | Right bank of the superior lateral sulcus (curvature and volume) | Right inferiorparietal cortex (area and volume) |
|  | Left paracentral cortex (volume) | Right entorhinal cortex (thicknessstd) |
|  | Left pars opercularis (area) | Left isthmus cingulate cortex (volume) |
|  |  | Right parahippocampal cortex (area) |
|  |  | Left supramarginal cortex (area) |
| DTI-FA | Splenium of corpus callosum (average) |  |
|  | Right posterior corona radiata (average) |  |

*Notes.* Features were identified via the SHAP (Shapley Additive exPlanations) technique (Lundberg and Lee, 2017) as described in Rane et al. (Rane et al., 2022) and are ordered in ascending order according to their importance. An exhaustive list of features and their corresponding SHAP values can be found in the porject’s GitHub repository (<https://github.com/RoshanRane/ML_for_IMAGEN>). Area, surface area; curvature, integrated rectified mean curvature; volume, gray matter volume; thickness, average thickness; thicknessstd, standard deviation of thickness.

## Results in a sample with maximum one BDE at age 14

In the exploration stage, counterbalancing was performed on 114 psychometric variables with at least 15% of cases in each group after binarization. When not controlling for any psychometric variable, the ML model controlling for sex and site achieved MBA_pre_ = 68.92 % (SD = 3.45 %) on the exploratory dataset with maximum one BDE at age 14. On the generalization dataset, the model prospectively predicted binge drinking at age 22 from sMRI features at age 14 with MBA_pre_ = 69.89 % (SD = 1.00 %; p = .008).





**Figure S1.** Exploration stage results: MBA_post-pre_ for psychometric variables in the exploratory dataset of adolescents with maximum one binge drinking episode at age 14 (*n* = 565). All values in percent. Vertical lines mark the threshold beyond which the lowest ~10% of the MBA_post-pre_ values are located. For visualization purposes, dot size increases with increasing absolute MBA_post-pre_. For more information on psychometric variables, see Table S1. Alcohol Problem Index, Alcohol Problem Index (White and Labouvie, 1989); AUDIT, Alcohol Use Disorder Identification Test (Saunders et al., 1993); BL, assessed at adolescent’s age 14; ESPAD, European School Survey Project on Alcohol and other Drugs; FU3, assessed at adolescent's age 22; LEQ, Stressful Life Event Questionnaire (Newcomb et al., 1981); MBA_pre_ / MBA_post_, mean balanced accuracy before / after performing *counterbalancing with oversampling* for each variable; TLFB, Timeline-Followback Interview (TLFB) (Sobell et al., 1996).





**Figure S2.** Generalization stage results: MBA_post_ for psychometric variables in the holdout dataset (*n* = 78). All values in percentage. Bonferroni-corrected *p*-palues denote if the MBA_(post-pre)_ per psychometric variable was significantly different from the mean permuted MBA_(post-pre)_ (Table S1, Supplementary Methods). Alcohol Problem Index, Alcohol Problem Index (White and Labouvie, 1989); AUDIT, Alcohol Use Disorder Identification Test (Saunders et al., 1993); BL, assessed at adolescent’s age 14; ESPAD, European School Survey Project on Alcohol and other Drugs; FU3, assessed at adolescent's age 22; LEQ, Stressful Life Event Questionnaire (Newcomb et al., 1981); MBA_post_, mean balanced accuracy after performing *counterbalancing with oversampling* for each variable; Timeline-Followback Interview (TLFB) (Sobell et al., 1996).

# REFERENCES

Cooper, M.L., 1994. Motivations for alcohol use among adolescents: Development and validation of a four-factor model. Psychol. Assess. 6, 117–128. https://doi.org/10.1037/1040-3590.6.2.117

Costa, P.T., McCrae, R.R., Psychological Assessment Resources, I., 1992. Revised NEO Personality Inventory (NEO PI-R) and NEO Five-Factor Inventory (NEO-FFI). Psychological Assessment Resources.

Herlin, B., Navarro, V., Dupont, S., 2021. The temporal pole: From anatomy to function—A literature appraisal. J. Chem. Neuroanat. 113, 101925. https://doi.org/10.1016/j.jchemneu.2021.101925

Humphreys, G.F., Jackson, R.L., Lambon Ralph, M.A., 2020. Overarching Principles and Dimensions of the Functional Organization in the Inferior Parietal Cortex. Cereb. Cortex 30, 5639–5653. https://doi.org/10.1093/cercor/bhaa133

Johns, P., 2014. Chapter 3 - Functional neuroanatomy, in: Johns, P. (Ed.), Clinical Neuroscience. Churchill Livingstone, pp. 27–47. https://doi.org/10.1016/B978-0-443-10321-6.00003-5

Jones, D.K., Williams, S.C.R., Gasston, D., Horsfield, M.A., Simmons, A., Howard, R., 2002. Isotropic resolution diffusion tensor imaging with whole brain acquisition in a clinically acceptable time. Hum. Brain Mapp. 15, 216–230. https://doi.org/10.1002/hbm.10018

Karlsson, J., Persson, L.-O., Sjöström, L., Sullivan, M., 2000. Psychometric properties and factor structure of the Three-Factor Eating Questionnaire (TFEQ) in obese men and women. Results from the Swedish Obese Subjects (SOS) study. Int. J. Obes. 24, 1715–1725. https://doi.org/10.1038/sj.ijo.0801442

Lorca-Puls, D.L., Gajardo-Vidal, A., PLORAS Team, Oberhuber, M., Prejawa, S., Hope, T.M.H., Leff, A.P., Green, D.W., Price, C.J., 2021. Brain regions that support accurate speech production after damage to Broca’s area. Brain Commun. 3, fcab230. https://doi.org/10.1093/braincomms/fcab230

Lundberg, S.M., Lee, S.-I., 2017. A Unified Approach to Interpreting Model Predictions, in: Guyon, I., Luxburg, U.V., Bengio, S., Wallach, H., Fergus, R., Vishwanathan, S., Garnett, R. (Eds.), Advances in Neural Information Processing Systems. Curran Associates, Inc.

Menardi, G., Torelli, N., 2014. Training and assessing classification rules with imbalanced data. Data Min. Knowl. Discov. 28, 92–122. https://doi.org/10.1007/s10618-012-0295-5

Molnar, C., 2022. Interpretable Machine Learning: A Guide for Making Black Box Models Explainable, 2nd ed.

Newcomb, M.D., Huba, G.J., Bentler, P.M., 1981. A Multidimensional Assessment of Stressful Life Events among Adolescents: Derivation and Correlates. J. Health Soc. Behav. 22, 400–415. https://doi.org/10.2307/2136681

Ojala, M., Garriga, G.C., 2010. Permutation tests for studying classifier performance. J. Mach. Learn. Res. 11.

Rane, R.P., de Man, E.F., Kim, J., Görgen, K., Tschorn, M., Rapp, M.A., Banaschewski, T., Bokde, A.L., Desrivieres, S., Flor, H., Grigis, A., Garavan, H., Gowland, P.A., Brühl, R., Martinot, J.-L., Martinot, M.-L.P., Artiges, E., Nees, F., Papadopoulos Orfanos, D., Lemaitre, H., Paus, T., Poustka, L., Fröhner, J., Robinson, L., Smolka, M.N., Winterer, J., Whelan, R., Schumann, G., Walter, H., Heinz, A., Ritter, K., IMAGEN consortium, 2022. Structural differences in adolescent brains can predict alcohol misuse. eLife 11, e77545. https://doi.org/10.7554/eLife.77545

Saunders, J.B., Aasland, O.G., Babor, T.F., De la Fuente, J.R., Grant, M., 1993. Development of the Alcohol Use Disorders Identification Test (AUDIT): WHO collaborative project on early detection of persons with harmful alcohol consumption-II. Addiction 88, 791–804. https://doi.org/10.1111/j.1360-0443.1993.tb02093.x

Smith, K.S., Tindell, A.J., Aldridge, J.W., Berridge, K.C., 2009. Ventral pallidum roles in reward and motivation. Behav. Brain Res. 196, 155–167. https://doi.org/10.1016/j.bbr.2008.09.038

Smith, K.W., Gierski, F., Andre, J., Dowell, N.G., Cercignani, M., Naassila, M., Duka, T., 2017. Altered white matter integrity in whole brain and segments of corpus callosum, in young social drinkers with binge drinking pattern. Addict. Biol. 22, 490–501. https://doi.org/10.1111/adb.12332

Smith, S.M., Jenkinson, M., Johansen-Berg, H., Rueckert, D., Nichols, T.E., Mackay, C.E., Watkins, K.E., Ciccarelli, O., Cader, M.Z., Matthews, P.M., Behrens, T.E.J., 2006. Tract-based spatial statistics: Voxelwise analysis of multi-subject diffusion data. NeuroImage 31, 1487–1505. https://doi.org/10.1016/j.neuroimage.2006.02.024

Sobell, L., Sobell, M., Buchan, G., Cleland, P., Fedoroff, I., Leo, G., 1996. The reliability of the Timeline Followback method applied to drug, cigarette, and cannabis use. Presented at the 30th Annual Meeting of the Association for Advancement of Behavior Therapy, New York, NY, US.

White, H.R., Labouvie, E.W., 1989. Towards the assessment of adolescent problem drinking. J. Stud. Alcohol 50, 30–37. https://doi.org/10.15288/jsa.1989.50.30

Woicik, P.A., Stewart, S.H., Pihl, R.O., Conrod, P.J., 2009. The substance use risk profile scale: A scale measuring traits linked to reinforcement-specific substance use profiles. Addict. Behav. 34, 1042–1055. https://doi.org/10.1016/j.addbeh.2009.07.001

Wyman, B.T., Harvey, D.J., Crawford, K., Bernstein, M.A., Carmichael, O., Cole, P.E., Crane, P.K., DeCarli, C., Fox, N.C., Gunter, J.L., Hill, D., Killiany, R.J., Pachai, C., Schwarz, A.J., Schuff, N., Senjem, M.L., Suhy, J., Thompson, P.M., Weiner, M., Jack Jr., C.R., Initiative, A.D.N., 2013. Standardization of analysis sets for reporting results from ADNI MRI data. Alzheimers Dement. 9, 332–337. https://doi.org/10.1016/j.jalz.2012.06.004

Zou, Y., Murray, D.E., Durazzo, T.C., Schmidt, T.P., Murray, T.A., Meyerhoff, D.J., 2018. White matter microstructural correlates of relapse in alcohol dependence. Psychiatry Res. Neuroimaging 281, 92–100. https://doi.org/10.1016/j.pscychresns.2018.09.004
